# Prediction of patient admission and readmission in adults from a Colombian cohort with bipolar disorder using artificial intelligence

**DOI:** 10.3389/fpsyt.2023.1266548

**Published:** 2023-12-21

**Authors:** María Alejandra Palacios-Ariza, Esteban Morales-Mendoza, Jossie Murcia, Rafael Arias-Duarte, Germán Lara-Castellanos, Andrés Cely-Jiménez, Juan Carlos Rincón-Acuña, Marcos J. Araúzo-Bravo, Jorge McDouall

**Affiliations:** ^1^Unidad de Investigación, Fundación Universitaria Sanitas, Psicopatología y Sociedad Research Group, Bogotá, Colombia; ^2^Fundación Universitaria Sanitas, Gerencia y Gestión Sanitaria Research Group, Instituto de Gerencia y Gestión Sanitaria (IGGS), Bogotá, Colombia; ^3^Psicopatología y Sociedad Research Group, Facultad de Medicina, Fundación Universitaria Sanitas, Bogotá, Colombia; ^4^Keralty, Bogotá, Colombia; ^5^University of Santander - UDES, Bucaramanga, Colombia; ^6^Computational Biology and Systems Biomedicine, Biodonostia Health Research Institute, San Sebastián, Spain; ^7^Ikerbasque, Basque Foundation for Science, Bilbao, Spain; ^8^Department of Cell Biology and Histology, Faculty of Medicine and Nursing, University of Basque Country (UPV/EHU), Leioa, Spain; ^9^Sanitas Crea Research Group, Fundación Universitaria Sanitas, Bogotá, Colombia

**Keywords:** bipolar disorder, electronic health records, machine learning, patient admission, patient readmission, risk factors

## Abstract

**Introduction:**

Bipolar disorder (BD) is a chronically progressive mental condition, associated with a reduced quality of life and greater disability. Patient admissions are preventable events with a considerable impact on global functioning and social adjustment. While machine learning (ML) approaches have proven prediction ability in other diseases, little is known about their utility to predict patient admissions in this pathology.

**Aim:**

To develop prediction models for hospital admission/readmission within 5 years of diagnosis in patients with BD using ML techniques.

**Methods:**

The study utilized data from patients diagnosed with BD in a major healthcare organization in Colombia. Candidate predictors were selected from Electronic Health Records (EHRs) and included sociodemographic and clinical variables. ML algorithms, including Decision Trees, Random Forests, Logistic Regressions, and Support Vector Machines, were used to predict patient admission or readmission. Survival models, including a penalized Cox Model and Random Survival Forest, were used to predict time to admission and first readmission. Model performance was evaluated using accuracy, precision, recall, F1 score, area under the receiver operating characteristic curve (AUC) and concordance index.

**Results:**

The admission dataset included 2,726 BD patients, with 354 admissions, while the readmission dataset included 352 patients, with almost half being readmitted. The best-performing model for predicting admission was the Random Forest, with an accuracy score of 0.951 and an AUC of 0.98. The variables with the greatest predictive power in the Recursive Feature Elimination (RFE) importance analysis were the number of psychiatric emergency visits, the number of outpatient follow-up appointments and age. Survival models showed similar results, with the Random Survival Forest performing best, achieving an AUC of 0.95. However, the prediction models for patient readmission had poorer performance, with the Random Forest model being again the best performer but with an AUC below 0.70.

**Conclusion:**

ML models, particularly the Random Forest model, outperformed traditional statistical techniques for admission prediction. However, readmission prediction models had poorer performance. This study demonstrates the potential of ML techniques in improving prediction accuracy for BD patient admissions.

## 1 Introduction

Bipolar disorder (BD) is a chronically progressive mental disorder with a prevalence that ranges from 1.1 to 2.4% (1, 2). BD is classified as type I if the patient has presented at least one manic episode, with or without depressive episodes, and as type II in the presence of at least one hypomanic episode, with no full manic episodes, and one major depressive episode (3). This condition is associated with a reduced quality of life and greater disability. Patients have been shown to have lower incomes, higher financial burdens, issues with social interactions and a greater overall frequency of use of health services (4–7). Furthermore, significant suicide attempt rates have been reported in patients with BD type I (36.3%) and BD type II (32.4%) (8, 9). Additionally, manic episodes give rise to destructive and reckless behavior secondary to an unstable mood, which in the long term can degrade cognitive functioning, interpersonal relationships, global functioning and social adjustment (10, 11).

Patient admissions are preventable events with a considerable impact on healthcare costs and a key quality metric for health systems around the world (12–14). Among psychiatric patients, whose disorders are characterized by chronicity and high recurrence rates, readmissions are of particular concern. The period immediately after a hospitalization is known to be a period of high risk for outcomes such as suicide and substance abuse relapse. Patient admission and readmission due to BD places a significant financial burden on medical services and caregivers (2, 15). Studies have reported relapse rates of up to 50% after 2 years in individuals receiving adequate psychopharmacological care (16). Self-monitoring data from the Sanitas Healthcare Management Organization (HMO) in Colombia show that in 2017, 319 patients with a BD diagnosis had a total of 427 hospital admissions (27% of the overall number of readmissions) with a mean hospital stay duration of 19 days.

The prediction of risk for patient admission and readmission may aid in disease management as well as in reducing the economic and social burdens caused by BD. Despite the considerable number of publications in the field of patient admission prediction, most studies address this issue in patients with non-psychiatric illness (17–21), substance abuse disorders, schizophrenia or postpartum depression (22–24). Prior work by Rotenberg et al. aimed to predict depressive relapses in patients with bipolar disorder using machine learning techniques, achieving F measures as high as 0.993 for a random forest model (25). Although certainly related, depressive relapses are only one potential form of relapse of BD and may indeed be less likely to require hospitalization than manic episodes. This is why this study developed prediction models for hospital admission/readmission within 5 years of diagnosis in patients with BD using ML techniques. With clinical data that is readily available in electronic health records, we show that random forest models are good predictors of both admission and time to admission in patients with BD.

## 2 Materials and methods

### 2.1 Study participants and design

Sanitas is a major Healthcare Management Organization (HMO) in Colombia with over 5 million patients under its care. Data used in this study were obtained from patients who received an incident diagnosis of BD (including both type I and type II patients) during outpatient visits to any of the Sanitas EPS healthcare facilities in Colombia during 2016. The patients were followed for 5 years after diagnosis until December 31st, 2020. The Sanitas EPS network includes high-complexity centers which offer inpatient mental health services, day hospitals, and priority and general psychiatric outpatient appointments. Patients were retrospectively included if they had an International Classification of Diseases 10 diagnostic code of F31 assigned to them at any of their outpatient visits during the study period. Subjects with a BD diagnosis were excluded if they required long-term hospitalization due to functioning or psychotic symptoms, or if their social context (poor social support) required them to remain hospitalized in mental health units despite their psychiatric condition no longer requiring hospitalization. This study followed the guidelines of the Colombian Ministry of Health resolution 8,430 of 1993, as well as the World Medical Association’s Declaration of Helsinki in its 2013 version, and the Council for International Organizations of Medical Sciences’ International Ethical Guidelines for Health-related Research Involving Humans. The protocol for this study was approved by the Research Ethics Committee of the Fundación Universitaria Sanitas (CEIFUS 341–19).

**Figure 1:**
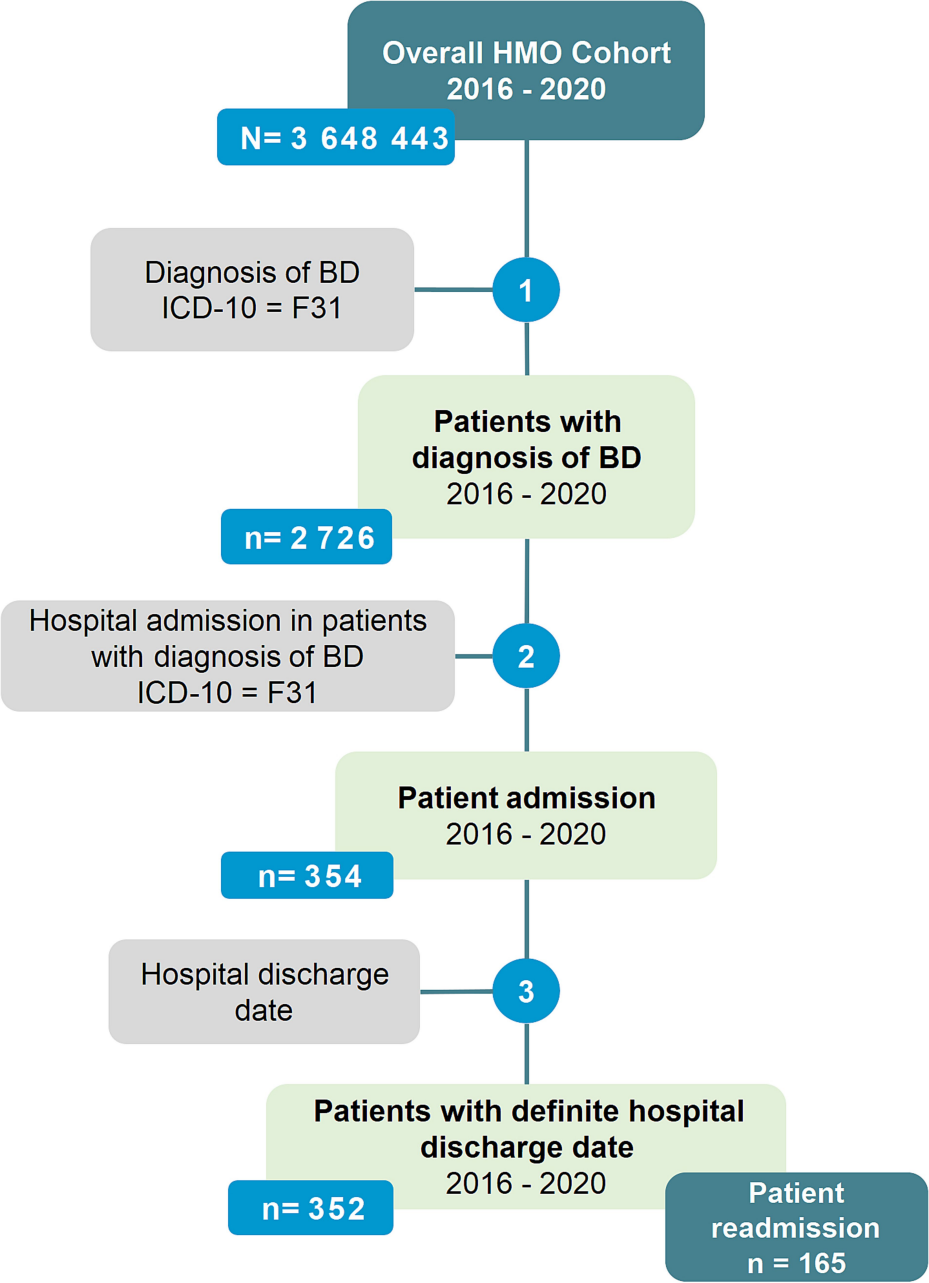
Patient flowchart.

Starting from an overall HMO patient cohort of over 3 million patients over the age of 18, we selected patients with an incident diagnosis of BD (diagnostic code F31), which totaled 2,726 patients. Of these, 354 patients had at least one psychiatric ward inpatient admission registered over 5 years. A complete record for the admission was available for 352 patients. These 352 patients comprised the admission dataset. Readmission occurred in 165 patients in whom a complete record for the readmission was available. The readmission dataset was obtained from these 165 patients (Figure 1).

### 2.2 Outcome definitions

The primary outcome in this study was a composite of hospital admission or readmission during the study period, although these outcomes were considered separately in model construction and selection. We selected this composite outcome as it is a key indicator of disease status in BD. An initial group of models aimed to predict a binary outcome of admission/readmission during the observation period, and a second group of survival models aimed to predict time to admission/readmission.

### 2.3 Candidate predictors

Candidate predictors for ML models were selected from features available in the standard electronic health record (EHR) and making use of established knowledge regarding risk factors for the study outcome. The initial set of extracted predictors included sociodemographic variables, and variables related to the psychiatric and medical history. Specifically, for the outcome of patient readmission a group of variables describing the characteristics of the prior hospitalization was included.

A total of 47 candidate variables were considered initially for the outcome of admission. However, since not all variables were plausibly related to the outcome of either admission or readmission (e.g., type of discharge cannot be used as a predictor), this led to variable exclusion and a reduced set of 29 variables. After completeness analysis, we excluded variables with missing data in more than 35% of the subjects, further reducing the set to 18 variables. The readmission dataset was refined from the same starting pool of variables. After an initial analysis, we were left with a reduced set of 33 variables (note that variables like “type of discharge” for the first admission can now be included). After completeness analysis, we were left with 21 predictor variables (Supplementary Table S1).

**Figure 2:**
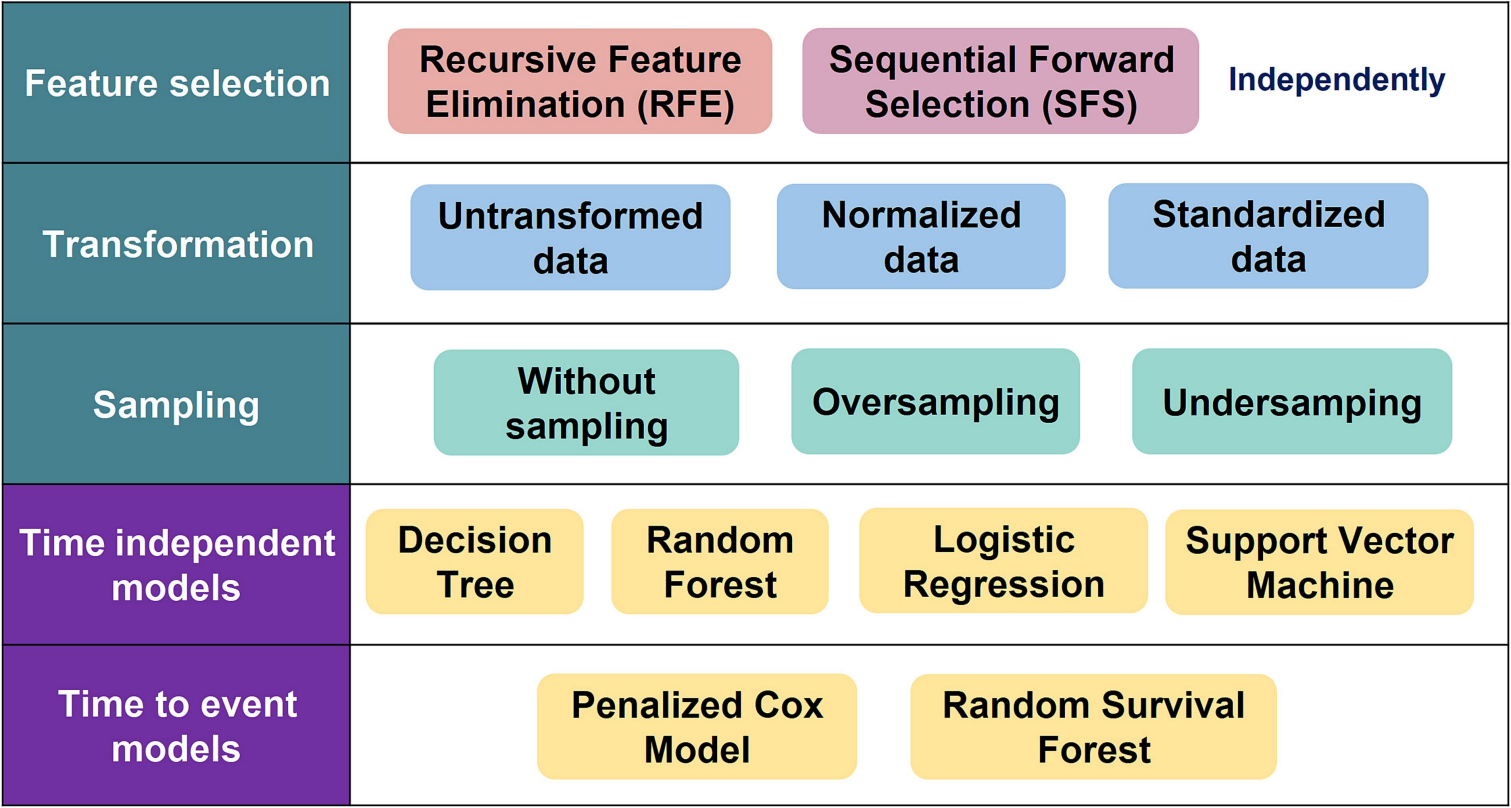
Study methods.

Variables with multiple categories were dichotomized into the presence or absence of their most frequent value prior to inclusion in the models but are presented in Table 1 as extracted from the EHRs. Continuous data were normalized/standardized and analyzed using correlation matrices. Variables were normalized by subtracting the minimum value of the variable and dividing the result by its range (difference between maximum and minimum). Standardized variables were the result of subtracting by the variables mean and dividing the result by their standard deviation. Although we planned to merge variables with correlations exceeding a pre-established threshold of 0.8, no such correlations were identified. Feature engineering made use of two techniques: Recursive Feature Elimination (RFE) and Sequential Feature Selection (SFS) (Figure 2). The RFE used a random forest to select features based on the top F1 scores, and the SFS used a sequential method to obtain a reduced set which minimized standard error and dimensionality (26, 27).

**Table 1:** Patient characteristics according to patient admission/readmission stratification.

Patient admission set of variables (18 variables)	Total (*N* = 2,726)	Admission (*n* = 354)	No admission (*n* = 2,372)	*p-*value
	n (%)			
Female	1747 (64.09)	229 (13.11)	1,518 (86.89)	0.846
Marital status				
Married	571 (20.95)	71 (12.43)	500 (87.57)	0.253*
Divorced	27 (0.99)	6 (22.22)	21 (77.78)	
Single	1902 (69.77)	256 (13.46)	1,646 (86.54)	
Common law marriage	209 (7.67)	20 (9.57)	189 (90.43)	
Widowed	17 (0.62)	1 (5.88)	16 (94.12)	
Healthcare regime				
Contributive	2,439 (89.47)	303 (12.42)	2,136 (87.58)	**<0.001**
Premium plan	189 (6.93)	42 (22.22)	147 (77.77)	
Subsidized	98 (3.60)	9 (9.18)	89 (90.82)	
Type of user				
Policyholder	1979 (72.60)	281 (14.2)	1,698 (85.8)	0.014
Dependent	747 (27.40)	73 (9.77)	674 (90.23)	
Residence (region)				
Bogota	1,024 (37.57)	304 (29.69)	720 (70.31)	**<0.001**
Caribbean	234 (8.58)	4 (1.71)	230 (98.29)	
Central	645 (23.66)	5 (0.78)	640 (99.22)	
Eastern	530 (19.44)	36 (6.79)	494 (93.21)	
Pacific	266 (9.76)	4 (1.5)	262 (98.5)	
Other	27 (0.99)	1 (3.7)	26 (96.3)	
Armed conflict victim	75 (2.75)	3 (4)	72 (96)	0.3
Socioeconomic status				
Low income	62 (2.27)	11 (17.74)	51 (82.26)	0.37
Middle income	2,637 (86.47)	341 (12.93)	2,296 (87.07)	
High income	27 (0.99)	2 (7.41)	25 (92.59)	
Borderline personality disorder features	113 (4.15)	37 (32.74)	76 (67.26)	**<0.001**
Hypertension	815 (29.9)	78 (9.57)	737 (90.43)	**<0.001**
COPD	115 (4.22)	16 (13.91)	99 (86.09)	0.872
Hepatitis	5 (0.18)	1 (20)	4 (80)	1
Hypothyroidism	618 (22.67)	116 (18.77)	502 (81.23)	**<0.001**
Two or more medical comorbidities	246 (9.02)	36 (14.63)	210 (85.37)	0.48

	Median (Q1-Q3)			*p-*value
Age (years)	52 (18–66)	45.5 (18–58)	53 (18–67)	**<0.001**
No. outpatient follow-ups	12 (0–25)	3 (0–14)	13 (0–26)	**<0.001**
Psychiatric emergency visits	0 (0–1)	1.0 (0–2)	0 (0–0)	**<0.001**
Medical emergency visits	0 (0–0)	0.0 (0–1)	0 (0–0)	0.354
No. hospitalizations due to medical conditions	0 (0–0)	0.0 (0–0)	0 (0–0)	0.586

Patient readmission set of variables (21 variables)	Total (*N* = 352)	Readmission (*n* = 165)	No readmission (*n* = 187)	*p-*value
	*n* (%)			
Female	228 (64.77)	112 (49.12)	116 (50.88)	0.301
Marital status				
Married	72 (20.45)	25 (34.72)	47 (65.28)	0.113*
Divorced	7 (1.99)	4 (57.14)	3 (42.86)	
Single	254 (72.16)	126 (49.61)	128 (50.39)	
Common law marriage	18 (5.11)	10 (55.56)	8 (44.44)	
Widowed	1 (0.28)	0 (0)	1 (100)	
Healthcare regime				
Contributive	304 (86.36)	139 (45.72)	165 (54.28)	0.529
Premium plan	38 (10.80)	21 (55.26)	17 (44.74)	
Subsidized	10 (2.84)	5 (50)	5 (50)	
Type of user				
Policyholder	283 (80.4)	136 (48.06)	147 (51.94)	0.444
Dependent	69 (19.6)	29 (42.03)	40 (57.97)	
Residence (region)				
Bogota	306 (86.93)	146 (47.71)	160 (52.29)	0.651*
Caribbean	4 (1.14)	3 (75)	1 (25)	
Central	4 (1.14)	2 (50)	2 (50)	
Eastern	34 (9.66)	13 (38.24)	21 (61.76)	
Pacific	3 (0.85)	1 (33.33)	2 (66.67)	
Other	1 (0.28)	0 (0)	1 (100)	
Armed conflict victim	3 (0.85)	2 (66.67)	1 (33.33)	0.602*
Socioeconomic status				
Low income	11 (3.12)	7 (63.64)	4 (36.36)	0.468*
Middle Income	338 (96.02)	156 (46.15)	182 (53.85)	
High Income	3 (0.85)	2 (66.67)	1 (33.33)	
Borderline personality disorder features	36 (10.23)	22 (61.11)	14 (38.89)	0.103
Hypertension	75 (21.3)	27 (36)	48 (64)	**0.046**
COPD	14 (3.98)	5 (35.71)	9 (64.29)	0.561
Hepatitis	1 (0.28)	0 (0)	1 (100)	1*
Hypothyroidism	119 (33.8)	59 (49.58)	60 (50.42)	0.54
Two or more medical comorbidities	37 (10.51)	15 (40.54)	22 (59.46)	0.521
Place of origin (placeholder)				
Outpatient clinic	6 (1.70)	2 (33,33)	4 (66,67)	0.48*
Emergency department	336 (95.45)	160 (47,62)	176 (52,38)	
Secondary transfer from other hospital	10 (2.84)	3 (30)	7 (70)	
Admission associated with manic episode	4 (1.14)	1 (25)	3 (75)	0.706
Cause of discharge				
Clinical improvement	326 (92.6)	152 (46,63)	174 (53,37)	0.91*
Voluntary discharge	16 (4.55)	9 (56,25)	7 (43,75)	
Escape	1 (0.28)	0 (0)	1 (100)	
Home hospital	3 (0.85)	1 (33,33)	2 (66,67)	
Transfer to another facility	6 (1.70)	3 (50)	3 (50)	

	Median (Q1-Q3)			*p-*value
Age (years)	48 (36–60)	47 (36–57)	49 (38–61)	0.108
No. outpatient follow-ups	2 (0–7)	1 (0–6)	3 (0–8)	**0.021**
Psychiatric emergency visits	0 (0–1)	0 (0–1)	0 (0–1)	0.078
Medical emergency visits	0 (0–0)	0 (0–0)	0 (0–0)	0.326
No. hospitalizations due to medical conditions	0 (0–0)	0 (0–0)	0 (0–0)	0.879

The *p-*value is calculated to address the statistical significance of the difference between the readmission and no readmission groups using the Chi-squared test (*Fisher’s exact test) or Mann–Whitney’s U test. COPD, Chronic Obstructive Pulmonary Disease. Bold values are statistically significant.

### 2.4 Sample size determination and sampling methods

Sample size was calculated based on the outcome of readmission since this was deemed to be the less frequent of the two outcomes in the composite. Sample size was estimated for the outcome of readmission assuming a Cox proportional hazards model with a hazard ratio of 1.8 and an overall readmission rate of 30%. Requiring a power of 90%, a significance level α of 5%, and a coefficient of determination of 0.3, we obtained a final sample size of 754 subjects, split between 174 readmissions and 580 controls.

Since the sample was heavily skewed toward patients with no admission, we made use of oversampling techniques to balance the classes and to facilitate prediction model training. Using a Synthetic Minority Oversampling Technique (SMOTE), in which synthetic data for patients with admission were generated, an oversampling set with 1,660 patients in each group was created (28). Additionally, an Edited Nearest Neighbor (ENN) method was used to generate an undersampling set which reduced the size of the larger non-admitted group, which generated a training set with 248 admitted and 1,479 non-admitted patients (29). The undersampling (US), oversampling (OS), and without sampling (WS) datasets were used to train models (Figure 2).

### 2.5 Statistical analysis methods

Means and standard deviations are presented for quantitative variables, and absolute and relative frequencies are presented for categorical variables. Normality was determined using both quantile-quantile plots and the Shapiro–Wilk test. All analyses were performed using Python software version 3.10.7 for Ubuntu version 20.04.5 LTS (Long Term Support).

#### 2.5.1 Logistic regression and machine learning algorithms

The data were randomly split into two sets in a 70:30 ratio using holdout validation, in which the training set was used to generate the prediction model for each algorithm and the test set was used to evaluate model performance. We used a more traditional statistical parametric model such as the Logistic Regression (LR) and three ML models to predict patient admission or readmission: Decision Trees (DT) (30), Random Forest (RF) (31), and Support Vector Machine (SVM) (32) (Figure 2). Each of these techniques has been applied in various ways in different mental disorders, including dementia, autism spectrum disorders, and obsessive compulsive disorder (33–35). Model performance was evaluated using the area under the receiver operating characteristic curve (AUC). Additionally, we report model accuracy, precision, recall and F1 score.

#### 2.5.2 Survival algorithms

Survival models were fitted to predict time to admission and first readmission. As with the ML models, data for survival model calibration were split into a training and test sets in a 70:30 ratio using holdout validation. Two survival models were used to estimate time to patient admission or readmission: one more standard such as the penalized Cox Model (P-Cox) (36) and a ML methods such as the Random Survival Forest (RSF) (37) (Figure 2). The performance of each model was evaluated using the concordance index (C-index), which is a generalization of the AUC which considers data censoring.

## 3 Results

### 3.1 RFE determined seven optimal variables for admission prediction

Regarding sex distribution, females were similarly common in either group (admission vs. no admission). Age was also a significant factor and admitted patients tended to be younger. The type of healthcare regime coverage showed a significant association with admission, with premium plan policy holders being most likely to be admitted. Patients from Bogotá were also more likely to be admitted than patients from the rest of Colombia. Armed conflict victim status did not differ significantly between admitted and non-admitted patients. Socioeconomic status, as systematically determined by the governmental statistical department, did not differ between groups (Table 1).

Concerning prior medical history, a history of hypothyroidism or hypertension was associated with admission. Admission was more likely in patients with borderline personality disorder features and in those with prior psychiatric emergency department visits. Interestingly, patients who were not admitted tended to have a greater number of outpatient follow-up visits (Table 1).

**Figure 3:**
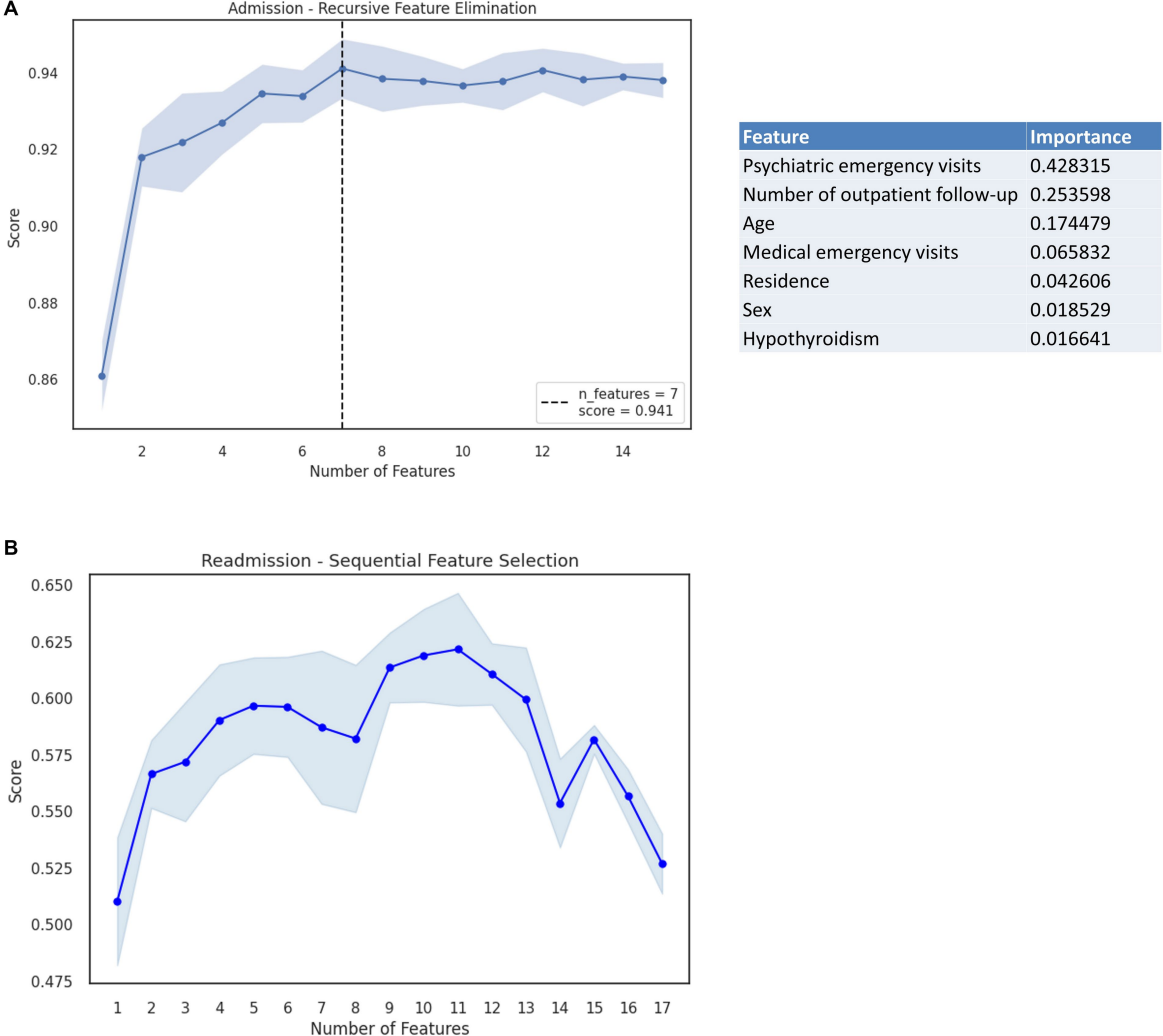
**(A)** RFE method considering the outcome of admission for BD and the importance analysis for each variable. **(B)** SFS method considering the outcome of readmission for BD (Shaded area corresponds to the standard error).

Feature engineering led to a reduced set of seven variables including psychiatric emergency visits, number of outpatient follow-ups, age, medical emergency visits, place of residence, sex, and history of hypothyroidism. Both the RFE and SFS methods were explored as alternatives for feature selection with the RFE method displaying better results. The RFE method displayed an F1 score of 0.941, compared with the F1 score of the SFS method of 0.939. Therefore, the RFE method was selected to determine the number of optimal variables (Figure 3A).

### 3.2 RF is the best model for patient admission prediction with an accuracy score of 0.951

**Figure 4:**
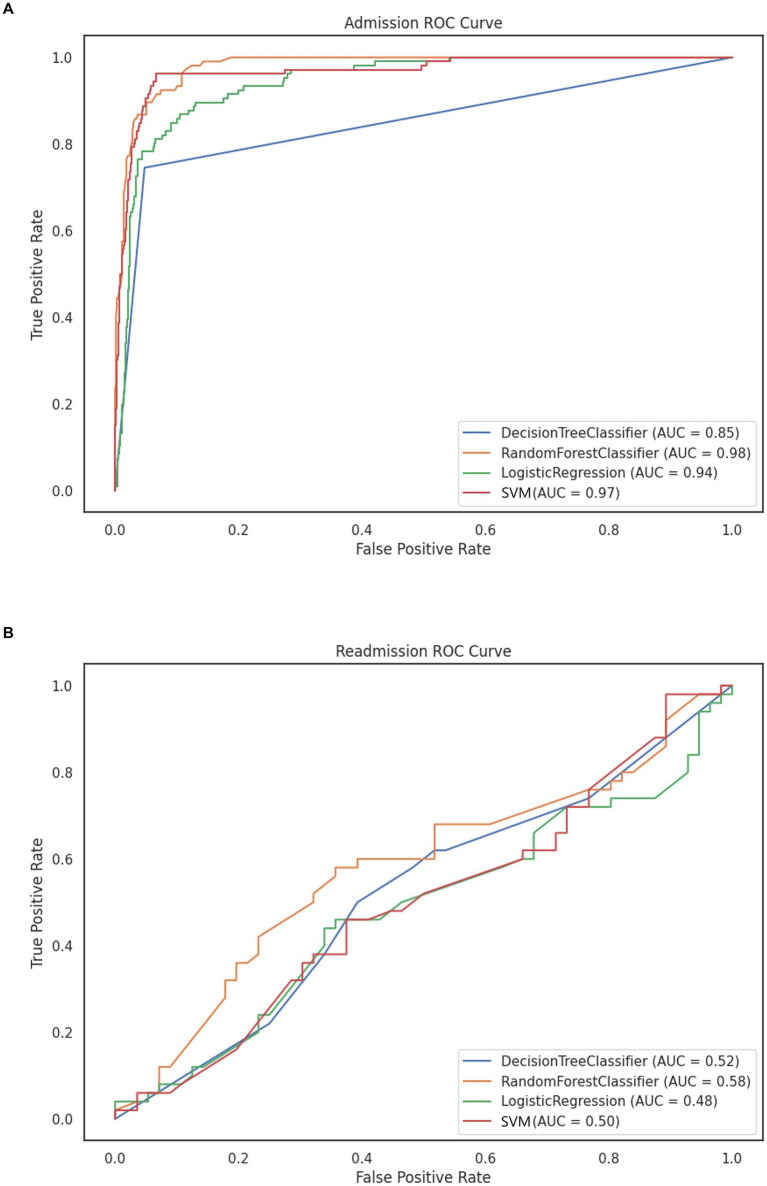
ROC curve of prediction models using **(A)** RFE and standardized data for BD admission without sampling. **(B)** SFS and normalized data for BD readmission.

Using the reduced set of variables to train models, the best performing model in this study was the Random Forest model, with an accuracy score of 0.951 and an AUC of 0.98 (Figure 4A). This best-performing model was trained using the starting dataset, without over or undersampling. The worst performing model was the Decision Tree model trained on the starting dataset (Table 2). We obtained similar results for the survival models for time to admission, where the random survival forest obtained the best results, with a C-index of 0.95 (the P-Cox model obtained a C-index of 0.897). The median follow-up for patients in the readmission group was of 52 months (IQR 40–57).

**Table 2:** Performance evaluation metrics for prediction of patient admission/readmission.

	Sampling type	Accuracy score	Precision Score	Recall score	F1 score	AUC
Patient admission						
Decision tree	WS	0.93	0.70	0.75	0.72	0.85
	OS	0.92	0.64	0.83	0.72	0.88
	US	0.91	0.60	0.92	0.72	0.91
Random forest	**WS**	**0.95**	**0.82**	**0.79**	**0.81**	**0.98**
	OS	0.94	0.72	0.87	0.79	0.98
	US	0.93	0.68	0.93	0.78	0.98
Logistic regression	WS	0.91	0.76	0.44	0.56	0.94
	OS	0.92	0.63	0.94	0.75	0.96
	US	0.93	0.70	0.80	0.75	0.96
Support vector machine	WS	0.95	0.80	0.79	0.80	0.97
	OS	0.92	0.62	0.97	0.76	0.98
	US	0.94	0.70	0.94	0.81	0.98
Patient readmission						
Decision tree	WS	0.55	0.52	0.62	0.56	0.52
Random forest	WS	0.55	0.52	0.60	0.56	0.58
Logistic regression	WS	0.55	0.52	0.46	0.49	0.48
Support vector machine	WS	0.52	0.49	0.48	0.49	0.50

Distribution type: without sampling (WS), oversampling (OS), undersampling (US). Best performing model in bold.

### 3.3 SFS determined 11 optimal variables for readmission prediction

Significant associations between study variables and the outcome of readmission were identified only for a prior medical history of hypertension and for the number of outpatient follow-ups (Table 1). Feature engineering led to a reduced set of 11 variables including psychiatric emergency visits, medical emergency visits, number of hospitalizations due to medical conditions, sex, type of user, place of residence, socioeconomic status, history of hypertension, history of Chronic Obstructive Pulmonary Disease (COPD), place of origin (placeholder), and cause of discharge. The SFS and RFE methods were considered in determining the optimal number of variables, ultimately obtaining a better performance under the F1 score metric for the SFS method (0.621 vs. 0.566) with the normalized data (Figure 3B).

### 3.4 Prediction of patient readmission is poor

Model performance for the outcome of patient readmission was poor, with the best performing model being again a RF (Table 2). None of the models, however, reached an AUC above 0.70 (Figure 4B). Performance for the time to readmission survival models was likewise poor, with the RSF reaching a C-index of 0.592 (the penalized Cox model obtained a C-index of 0.497). The median follow-up time for patients in this group was of 18 months (IQR 6–35).

## 4 Discussion

The prediction of patient admissions in any chronic disease is of great importance to researchers, public health planners and administrators (38, 39). Due to the varying features of different health systems, it is necessary to obtain different prediction tools for each population and health system to provide adequate risk management. To the best of our knowledge, this is the first study aiming to predict this outcome in a sample of subjects with BD in Colombia. We studied the outcomes of admission and readmission in subjects with BD in a large sample of 2,726 patients in Colombia, obtaining models with excellent predictive performance for the outcome of admission.

The ML models used in this study outperformed traditional statistical techniques (LR and P-Cox) in all cases with the random forest being the superior model in all cases. However, when comparing the results for the outcome of admission the random forest is only marginally superior to the LR model (AUC 0.98 vs. 0.94, respectively), which has the advantage of the latter of providing an easily interpretable model. This interpretability may be of critical importance in decision making. The difference between traditional statistical models and ML became more evident in the survival models for admission, in which the P-Cox model achieved a C-index of 0.897, while the SRF reached a C-index of 0.95. This difference between traditional statistical models and ML models was still evident in the survival models for readmission, though both models had very poor performance. This poor performance is likely due to the intrinsic difficulties associated with the prediction of survival (no readmission) compared with merely predicting the occurrence of the event within a given time frame. In addition, holdout validation was used to split the dataset corresponding to this outcome, which contained a smaller number of patients, leading to less precise estimates.

Random forest models are almost always superior to individual decision trees due to a variety of reasons. First, the random forest combines multiple decision trees which allows for a reduced variance and over-adjustment inherent to isolated trees. More robust and generalizable models can be derived from the averaging of several decision trees. Furthermore, the random forest models can also capture non-linear relationships and interactions between features. Lastly, random forest models can efficiently handle large datasets with high dimensionality, which makes them an adequate solution to complex problems like the prediction of BD admissions and readmissions.

All of the models explored for the outcome of admission had good to excellent performance, which was in contrast to the readmission models. ML models are known to require larger datasets than traditional statistical methods in order to produce better relative performance, and despite over and undersampling, the training dataset for readmission was considerably smaller (40). Prospective validation for these models is also lacking and it is an area for further research. The results suggested that ML analytics has the potential to provide risk calculators to aid in predicting clinical prognosis (including patient admissions), for individual patients (41).

Two prior studies have aimed to predict admission or readmission in BD patients. The study by Salem et al. aimed to predict readmission within 30 days after inpatient treatment of patients with a Diagnostic and Statistical Manual of Mental Disorders (DSM)-IV diagnosis of BD using a SVM technique. Importantly, this study relied on features extracted from the Borderline Personality Questionnaire (BPQ), the scores of which were available for all subjects. The study found good discriminative ability with an area under the Receiver Operating Curve (ROC) of 0.86, concluding that borderline personality features, as measured using the BPQ, were good predictors of early readmission. The external validity of this study is limited by the availability of data on its primary predictor, which is not a standard feature of electronic health records of patients with BD (42). A second study, by Edgcomb et al., also aimed to predict risk of readmission after 30 days in patients with BD. This study took into consideration standard data from EHRs, not including any kind of standardized measurement. Using classification trees, this model achieved high accuracy with an area under the ROC curve of 0.88. An additional strength of this study was the interpretability of the model which can be easily adapted into medical thinking (43).

In this study, the presence of borderline personality disorder features was significantly associated with admission in our first analyses. Prior work has identified these features as being a key predictor in rapid readmissions in patients with BD (42). Although this variable was not significantly associated with readmission, it did meet the requirements for inclusion as a feature in the predictive models. Similarly, some medical comorbidities were both significantly associated with admission (hypertension, hypothyroidism), and met criteria for inclusion in the readmission models. This highlights their importance in considering the risk of admission/readmission in patients with BD.

A number of clinical variables related to the patient’s mood, sleep quality, self-reported energy levels and medication adherence were not available to us, and they are known to be good predictors of both depressive and manic episodes in BD (43, 44). This was probably due to the paucity of information registered in EHRs which is more likely to contain specific changes in the disease’s symptoms and signs throughout clinical follow-up, instead of the evolution of all the clinical variables considered.

In obtaining the final sample, we were significantly limited by the availability of information. Despite having access to a larger cohort of patients with BD, data availability in EHRs limited their inclusion in the study. Both the number of registries and the information contained within them may have varied systematically. Physicians may be less inclined to describe clinical features in detail for patients who they deem low risk. Patients at greater risk of admission may also be those with poor adherence to outpatient follow-up, leading to fewer records from which to draw information. This may have led to selection bias in our sample.

Future work will concentrate on the prospective validation of the models obtained in this project, including the use of cross-validation methods (e.g., K-fold, leave one out) to allow more robust estimates of model performance by evaluating the model on different combinations of data. The limitations caused by the scarcity of data could be mitigated by EHR systems which can more intuitively allow the psychiatrist to rapidly register certain features of the mental exam, enabling more comprehensive work in mental health to be carried out. Additionally, the incorporation of longitudinal data, genetic analysis and dynamic modeling could facilitate the development of personalized treatment strategies that account for individual variations in disease progression and response to interventions in BD.

## 5 Conclusion

This study highlights the potential of utilizing ML techniques to predict hospital admission and readmission in patients with BD. The results demonstrate that ML models, particularly the Random Forest algorithm, exhibit superior predictive performance compared to traditional statistical methods. By leveraging EHRs and incorporating a range of sociodemographic and clinical variables, these models provide valuable insights into the factors influencing hospitalization in BD patients.

The use of ML techniques in psychiatric research, particularly in the context of BD, has the potential to deepen our understanding of the underlying mechanisms and pathophysiology of the condition. By uncovering novel associations and risk factors for patient admissions, these models contribute to the ongoing efforts to unravel the complexities of BD and guide future research directions.

## Data availability statement

The datasets presented in this article are not readily available due to patient confidentiality and data protection. The raw data supporting the conclusions of this article will be made available by the corresponding author only if this request is approved by the Research Ethics Committee of Fundación Universitaria Sanitas, Bogotá D.C., Colombia, following patient protection regulations in Colombia. Requests to access the datasets should be directed to the corresponding author.

## Ethics statement

The studies involving humans were approved by Comité de Ética en Investigación Fundación Universitaria Sanitas (CEIFUS). The studies were conducted in accordance with the local legislation and institutional requirements. Written informed consent for participation was not required from the participants or the participants’ legal guardians/next of kin in accordance with the national legislation and institutional requirements.

## Author contributions

MP-A: Conceptualization, Data curation, Formal analysis, Funding acquisition, Investigation, Methodology, Software, Supervision, Validation, Visualization, Writing – original draft, Writing – review & editing, Project administration. EM-M: Data curation, Formal analysis, Investigation, Methodology, Software, Validation, Visualization, Writing – original draft, Writing – review & editing. JMu: Data curation, Formal analysis, Investigation, Methodology, Software, Validation, Visualization, Writing – original draft, Writing – review & editing. RA-D: Data curation, Investigation, Methodology, Validation, Visualization, Writing – original draft, Writing – review & editing. GL-C: Conceptualization, Data curation, Investigation, Supervision, Visualization, Writing – original draft, Writing – review & editing. AC-J: Conceptualization, Data curation, Formal analysis, Supervision, Visualization, Writing – review & editing. JR-A: Conceptualization, Data curation, Formal analysis, Methodology, Supervision, Validation, Visualization, Writing – review & editing. MA-B: Conceptualization, Investigation, Methodology, Resources, Supervision, Validation, Visualization, Writing – review & editing. JMc: Conceptualization, Funding acquisition, Investigation, Methodology, Project administration, Resources, Supervision, Visualization, Writing – review & editing.
